# Antimicrobial Pharmacokinetic Considerations in Extracorporeal Membrane Oxygenation

**DOI:** 10.3390/jcm13123554

**Published:** 2024-06-18

**Authors:** Kevin Johns, Gregory Eschenauer, Angela Clark, Simona Butler, Sabrina Dunham

**Affiliations:** 1 Department of Pharmacy Services, University of Michigan Health, Ann Arbor, MI 48109, USA; gregorye@med.umich.edu (G.E.); aclar@med.umich.edu (A.C.); simonao@med.umich.edu (S.B.); 2 Department of Clinical Pharmacy, University of Michigan College of Pharmacy, Ann Arbor, MI 48109, USA

**Keywords:** extracorporeal membrane oxygenation, critical illness, antimicrobials, pharmacokinetics

## Abstract

Critical illness creates challenges for healthcare providers in determining the optimal treatment of severe disease, particularly in determining the most appropriate selection and dosing of medications. Critically ill patients experience endogenous physiologic changes that alter the pharmacokinetics (PKs) of medications. These alterations can be further compounded by mechanical support modalities such as extracorporeal membrane oxygenation (ECMO). Specific components of the ECMO circuit have the potential to affect drug PKs through drug sequestration and an increase in the volume of distribution. Factors related to the medications themselves also play a role. These PK alterations create problems when trying to properly utilize antimicrobials in this patient population. The literature seeking to identify appropriate antimicrobial dosing regimens is both limited and difficult to evaluate due to patient variability and an inability to determine the exact role of the ECMO circuit in reduced drug concentrations. Lipophilic and highly protein bound medications are considered more likely to undergo significant drug sequestration in an ECMO circuit, and this general trend represents a logical starting point in antimicrobial selection and dosing in patients on ECMO support. This should not be the only consideration, however, as identifying infection and evaluating the efficacy of treatments in this population is challenging. Due to these challenges, therapeutic drug monitoring should be utilized whenever possible, particularly in cases with severe infection or high concern for drug toxicity.

## 1. Critical Illness Effects on Drug Pharmacokinetics

Critical illness presents challenges to healthcare providers in determining both the cause of and the most optimal treatment for severe disease. Medication selection coupled with optimal dosing is essential for treatment success. Patients who are critically ill, however, experience endogenous physiologic changes that influence the way medications are absorbed, metabolized, and eliminated from the body [1]. These pharmacokinetic (PK) parameters can be influenced by organ dysfunction, volume derangements, and the acute phase response to illness [1]. Interventions to treat illness should not be discounted as mechanical support modalities, such as hemofiltration or extracorporeal membrane oxygenation (ECMO), can also play a role in PK alterations seen in critical illness [2].

Absorption is the first step in the drug PK process that can be affected by critical illness. Many medications are absorbed in the gut, and during the acute phase response, blood is shunted away from the gastrointestinal (GI) tract towards vital organs such as the brain and heart [1]. This shunting of blood, along with potential gut dysmotility, reduces gut perfusion and the overall absorption of medication in the GI tract [3]. This effect is compounded with the use of vasoactive agents that can further decrease blood flow to the gastrointestinal tract [1]. Nutritional status may also affect drug absorption. Normal GI function is maintained through food passing through the gut, and a lack of enteral feeding leads to intestinal atrophy and decreased GI surface area, consequently decreasing drug absorption [4]. Critically ill patients often go long periods of time without enteral feeding, whether it be for procedures, issues with oral access, or an inability to tolerate enteral feeds, placing them at high risk for GI malabsorption [1]. Further exacerbating the issue is the use of medications that delay gastric emptying, particularly opioids commonly used in critical illness, and this may contribute to reduced drug absorption [5]. Lastly, these patients, like all patients, are at risk of drug–drug interactions that may further contribute to reduced drug absorption.

Drug distribution is also affected during critical illness. Changes in pH can affect drug penetration into tissues [1]. An overall deficiency in serum proteins can also lead to decreased drug distribution as protein is responsible for a large amount of drug binding and transportation [1]. The primary driver of altered distribution in the critically ill population, however, is variable volume of distribution [1]. These patients will often experience large shifts in volume status as a result of resuscitation and third spacing of fluids during systemic inflammation [1]. Serum drug concentration is directly related to the administered dose and volume of distribution ($C=\frac{Dose}{Volume\;of\;distribution}$), so any augmentation in the volume of distribution will decrease the drug concentration when giving equivalent doses [1].

Drug metabolism is an important step in pharmacokinetics that impacts treatment response and elimination. Some medications require metabolism to become active while others require metabolism to be broken down, removed from circulation, and prepared for elimination [1]. For example, clopidogrel is metabolized to its active metabolite by the CYP2C19 enzyme, a conversion that is necessary for clopidogrel to exert its pharmacologic effect [6]. Metabolism, like other PK stages, has the potential to be affected by critical illness. The liver is the epicenter of drug metabolism in the body, and any alteration in blood supply to the liver can limit its ability to function properly [7]. A reduced blood supply, common in critical illness due to blood loss or shunting, can also restrict the amount of medication ready for metabolism that is delivered to the liver [1]. Additionally, the body’s stress response leads to the release of proinflammatory cytokines that have been shown to inhibit cytochrome P450 enzymes, which are a key component of phase I metabolism [7]. Whatever the case, critical illness can reduce drug metabolism, creating opportunities for both ineffective therapy and the potential for toxicities through alterations in serum concentrations.

Lastly, drug elimination is also altered during critical illness, although variations in excretion can result in both increased and decreased serum concentrations. Just like metabolism is highly dependent on liver function, elimination is largely dependent on kidney function as renal elimination is the principle means of drug elimination [1]. Like all organs, the kidneys rely on adequate cardiac output and perfusion, which may be augmented by initial resuscitative efforts, increasing drug elimination [1]. Any decrease in renal blood supply, which is common in critical illness, would then lead to reduced elimination and increased serum concentrations of drug metabolites [8]. Kidney function, and therefore drug excretion, can be widely variable during the different stages of critical illness, making close monitoring of surrogate markers (e.g., serum creatinine, urine output, etc.) imperative.

## 2. ECMO Effects on Drug Pharmacokinetics

Extracorporeal membrane oxygenation (ECMO) is a type of mechanical support modality that can provide both respiratory and cardiac support, depending on the individual needs of a patient [9]. Two common configurations are veno-venous (VV) ECMO for respiratory support or veno-arterial (VA) ECMO for respiratory and cardiac support, although other complex configurations are possible [9]. In all cases, ECMO serves as a temporary treatment bridge to recovery or until a more permanent management strategy can be implemented [9]. ECMO provides support by draining deoxygenated blood from venous circulation via a large-bore cannula, pumping it into an oxygenator where mechanical gas exchange takes place, and returning it to circulation (venous or arterial) via a second cannula [10].

The components of the ECMO circuit used along with the possibility of multiple configurations create the opportunity for PK alterations, added to the baseline changes resulting from the patient’s critical illness [2]. Factors that influence PK differences in this setting can be attributed to individual components of the ECMO circuit through drug sequestration and an increased volume of distribution [2].

The cannulas (tubing) and oxygenator within the ECMO circuit are largely responsible for drug removal [2]. Each component increases the surface area for drug binding, with the tubing playing a much larger role than the oxygenator [2]. However, the type of oxygenator membrane used in the circuit, along with the coating of the oxygenator, may impact the degree of drug sequestration, as polymethylpentene and other membranes provide a more durable coating that is more resistant to leakage and may reduce the amount of drug lost in the circuit [11]. The type and age of the circuit can also influence the amount of drug sequestration [12]. Circuit tubing is manufactured as both modified and non-modified, with the latter comprising traditional polyvinyl chloride or diethylphenyl phthalate tubing without additional additives [13]. Modified tubing, on the other hand, refers to circuits that utilize other components (e.g., heparin, albumin, and other synthetic polymers) in addition to the traditional coating seen in non-modified tubing [13,14]. The current understanding of drug sequestration suggests that modified circuit tubing sequesters more medication than non-modified tubing, although the exact mechanism for differences in drug absorption remains unclear [13]. These coatings, however, have the tendency to degrade over time, which may affect the amount of drug sequestration seen over the life of the circuit. In contrast, a newer ECMO circuit is believed to sequester more drug when compared to a circuit used for multiple days [12]. As drug continuously binds to the tubing and other components over the life of the circuit, the surface area needed for additional drug sequestering is limited, creating a theoretical plateau in the amount of drug that is lost [2]. Circuit changes would again increase the available surface area, which may potentially impact medication dosing and efficacy.

Circuit tubing and the priming solution of the circuit can also contribute to PK derangements by increasing the apparent volume of distribution [2]. Circuit tubing adds to the surface area while the priming solution adds to the physical volume of the circulating fluid (a typical average volume of anywhere from 100 mL to 1000 mL depending on provider preference and patient population) [15]. Different types of priming solutions include crystalloids, colloids, and those with added electrolytes or other contents that have questionable impact on drug sequestration on their own [12]. Of the ECMO circuit factors, the volume of distribution added by the circuit is likely mostly related to the surface area added by the cannulas [2].

The ECMO modality may also affect drug PKs, although this concept may not be well elucidated. The key feature of the ECMO modality in this context is the pulsatility of the blood flow [16]. VV ECMO, by means of returning blood to the venous circulation, utilizes native cardiac function, resulting in a pulsatile flow similar to a normal heartbeat [9]. VA ECMO, on the other hand, returns blood to the arterial circulation and bypasses the heart altogether, which results in non-pulsatile flow [9]. Historical data suggest that non-pulsatile flow may lead to a reduction in renal cortical blood flow through renin–angiotensin activation, affecting urine output and volume [16,17,18,19]. Conversely, renal perfusion pressure is known to improve through VA ECMO augmenting urinary output [20]. It is unclear how this dichotomy interplays relative to the impact on medication metabolism.

## 3. Medication Factors in Altered Drug Pharmacokinetics

Like alteration factors related to the ECMO circuit, there are a number of medication-specific factors that may lead to drug sequestration in the circuit [2]. In particular, a medication’s lipophilicity or hydrophilicity, protein binding, volume of distribution, molecular size, and molecular charge will influence the amount of drug sequestration [2,8]. Of these PK characteristics, a drug’s lipophilicity (indicated by its LogP value) and degree of protein binding will influence both its volume of distribution and drug sequestration to the greatest degree [8,21]. Hydrophilic medications have lower volumes of distribution compared to lipophilic medications, which have higher volumes of distribution [8]. Typically, medications that are highly lipophilic (with a high LogP), highly protein bound, and are large-sized molecules with a charge will be subject to a higher degree of drug sequestration (Table 1) [2]. Conversely, smaller, uncharged molecules that are hydrophilic (low LogP) with low protein binding tend to undergo minimal sequestration, if any (Table 1) [2].

For context, LogP is the measure of a molecule’s preference to dissolve in either water or an organic solvent (i.e., the partition coefficient between 1-octanol and water) and is an indicator of a molecule’s lipophilicity or hydrophilicity [21]. When looking at an individual LogP for a specific medication, lower values (less than 1) are reflective of a hydrophilic molecule while higher values (greater than or equal to 1) indicate a lipophilic molecule (Table 1) [2]. Protein binding is typically listed in percentages and designated as high, moderate, or low protein binding [2]. Percentage cutoffs may differ depending on the literature source, but in general, highly protein bound medications have percentages greater than or equal to 50% (Table 1) [2].

## 4. Antimicrobial Considerations in ECMO

Critically ill patients are at high risk of infection. Due to the factors described above, antimicrobial exposures vary considerably in this population [22]. In one prospective, multinational study of patients in the ICU receiving antimicrobials, inter-patient beta-lactam trough concentrations varied by up to 100-fold [22]. For patients requiring ECMO support, variables such as the potential for significant sequestration further increase the potential for unpredictable antimicrobial target attainment [2]. This increases the potential for the development of resistance, toxicity, and/or inadequate treatment of severe infection [22]. Accordingly, it is crucial to identify strategies to guide the safe and effective use of antimicrobials in patients on ECMO.

There is a growing literature base that has sought to provide possible answers to this question by examining the pharmacokinetics of antimicrobials in patients on ECMO to identify optimal dosing strategies. Understandably, however, such data remain limited for several reasons. First, given the nature of ECMO (e.g., variability in number of pump runs, duration of therapy, patient indication/prognosis, etc.) and the complexity of care of patients receiving ECMO, much of the literature consists of case reports or small case series. These are limited by the lack of a control (non-ECMO) group. In addition, given the known substantial PK variability in critically ill patients, it is difficult to interpret the independent effect of the circuit. Secondly, as described previously, the effects of ECMO on drug sequestration are not static over time, and thus, even serial sampling early in therapy may not adequately represent exposures later in the life of the tubing. Finally, even studies with a non-ECMO control group will demonstrate substantial inter-patient variability.

This does not necessarily mean that no strategies exist to inform the optimal dosing of antimicrobials in patients receiving ECMO. First, concerns regarding inadequate exposures resulting from drug sequestration, increased volume of distribution, high-flow rate CRRT, and other factors are most relevant in the treatment of infections caused by organisms with elevated minimum inhibitory concentrations. Confident determination of infection is often challenging, and a substantial proportion of ECMO patients receiving antimicrobials are likely not infected. Furthermore, many patients are infected with rather susceptible organisms, and so studies that index exposures to MIC breakpoints will overstate the rate of suboptimal therapy [23]. However, for those patients with serious infections caused by more resistant organisms, it is likely that individual-patient therapeutic drug monitoring may be the only way to confidently manage drug choice and dosing [21]. Finally, acknowledging the uncertainties surrounding the literature, an overall approach based on an antimicrobial’s PK characteristics represents a reasonable starting point when attempting to adequately dose these medications in patients receiving ECMO support. Table 2 summarizes the PK characteristics of antimicrobials commonly used in critical illness. The majority of these anti-infectives are thought to follow the general trend that lipophilic medications with higher protein binding undergo a higher degree of drug sequestration in the ECMO circuit (Table 1) [2]. By understanding these mechanisms of PK alterations in ECMO and medication-specific factors that may predispose a drug to sequestration, clinicians may be able to better predict optimal drug selection and appropriate dosing schemes and drug monitoring [24].

As an example, piperacillin–tazobactam is a broad-spectrum combination beta-lactam/beta-lactamase inhibitor used frequently as an antipseudomonal agent in critically ill patients [25]. It has a predicted LogP value of 0.67 (indicating hydrophilicity) and a protein binding percentage of 30%, which does not portend a high degree of drug sequestration [25]. An observational, prospective, multicenter, case-control study comparing 42 patients receiving piperacillin–tazobactam [21 ECMO (17 of which were on VA ECMO) and 21 non-ECMO] found no effect of ECMO on serum drug concentrations [26]. Non-ECMO patients were matched in a 1:1 ratio with ECMO patients based on each individual’s creatinine clearance and SOFA severity score [26]. Serial trough concentrations were drawn according to the dosing regimen, and there was no difference in the proportion of time piperacillin concentrations were deemed adequate based on a pre-defined trough goal of ≤ 64 mg/L [26]. Similar findings have been reported for cefepime, another broad-spectrum antipseudomonal antibiotic used in critical illness [27,28].

One thought provoking case report further appears to support the standing that understanding drug PK characteristics is helpful in optimizing the treatment of infections in ECMO patients. Amphotericin is an antifungal used to treat severe fungal infections in the ICU. This agent has two different formulations that differ greatly in their PK profiles. Liposomal amphotericin B, as the name suggests, is a lipophilic formulation [29]. The deoxycholate formulation of amphotericin, on the other hand, is also highly protein bound (greater than 95%) but is more hydrophilic, with a predicted LogP of 0.8 [30]. The presumed drug sequestration of amphotericin by the ECMO circuit differs depending on the formulation used [31]. A case report of a 50-year-old male treated with amphotericin for disseminated blastomycosis shows these differences [31]. The patient was started on liposomal amphotericin B dosed at 5.1 mg/kg/day [31]. The day after starting treatment, the patient required VV ECMO cannulation and continuous renal replacement therapy (CRRT) [31]. A serum amphotericin level on day 4 of therapy was undetectable, and the patient was changed to the deoxycholate formulation the following day, dosed at 1 mg/kg/day [31]. The resulting level after the switch was 3.8 mcg/mL [31]. Based on the results of the report, amphotericin B deoxycholate may be the preferred formulation of amphotericin in patients on ECMO, and drug level monitoring, when possible, is recommended.

Recognizing the limitations of studies in this setting, each of these serve as examples of instances that follow the proposed drug sequestration trend. Both piperacillin–tazobactam and cefepime are hydrophilic molecules with low amounts of protein binding, and the serum concentrations of each medication were not noted to be affected by ECMO cannulation [26,28]. Likewise, the liposomal formulation of amphotericin B, which is lipophilic and highly protein bound, experienced enough drug sequestration that a drug level was undetectable [31]. Once the formulation was adjusted to the deoxycholate formulation, which is highly protein bound but hydrophilic, drug sequestration was not as profound and drug levels improved [31].

However, a case from our institution highlights the overarching rule that while our current understanding of drug characteristics on ECMO are helpful, they remain fallible. Posaconazole is an azole antifungal agent that is used to treat severe fungal infections such as invasive pulmonary *Aspergillus* [32]. It is lipophilic (predicted LogP of 5.4) and highly protein bound (greater than 98%) [32]. Posaconazole would thus be predicted to be subject to high drug sequestration in an ECMO circuit based on its PK characteristics. Here, we report a local case involving a 58-year-old male who underwent bilateral lung transplantation and received posaconazole for prophylaxis against *Aspergillus*. The patient was initiated on VV ECMO the day after transplant (post-operative day 1) and started on posaconazole 300 mg daily the following day (post-operative day 2). He was decannulated 3 days after starting posaconazole therapy (post-operative day 5). An initial posaconazole trough concentration on day 7 of therapy was therapeutic at 888 ng/mL (goal > 700 ng/mL per protocol). On day 14 of posaconazole therapy, the patient was re-cannulated to restart VV ECMO. A second therapeutic posaconazole trough concentration was obtained one week later (day 21 of therapy) and resulted in a concentration of 1360 ng/mL. Approximately 1 month after beginning posaconazole, the patient decompensated further, requiring initiation of CRRT and placement of an additional ECMO cannula due to the need for increased ECMO flows. The patient remained on CRRT and ECMO support for the duration of his treatment course. Due to the concern for variable posaconazole concentrations given the concurrent use of ECMO and CRRT and the rapid changes in the patient’s volume status, a third posaconazole trough concentration was obtained (on day 37 of therapy) of 174 ng/mL, which was significantly lower than the previous levels. The posaconazole dose was increased to 300 mg twice daily, and a level was obtained 4 days later, which was therapeutic at 1400 ng/mL. Another surveillance trough concentration was drawn approximately 2 months after the dose change and remained therapeutic at 1720 ng/mL. The patient did remain without a fungal infection but unfortunately further decompensated and passed away approximately 3 months post-transplant.

This posaconazole case presents some conundrums based on what is known about posaconazole’s lipophilicity and high protein binding [32]. Generally, posaconazole would be expected to undergo significant drug sequestration in the ECMO circuit. In this case, however, an initial posaconazole trough concentration was therapeutic and similar to a pre-ECMO level, suggesting little to no drug loss in the circuit. However, after the addition of another ECMO cannula, the trough concentration decreased significantly. Presumably, additional ECMO tubing would potentiate an increase in drug sequestration, and additional cannulas would further increase the available surface area for the sequestration of medications in the circuit, all of which can potentially lead to reduced serum concentrations [2]. The timing and degree of the posaconazole level decrease suggests possible drug sequestration in the ECMO circuit, and more emphatically demonstrates the unpredictability of drug sequestration and serum concentrations in this patient population.

## 5. Conclusions

Critically ill patients receiving ECMO support are complicated and optimal dosing of antimicrobials in these patients is challenging. Utilizing LogP and protein binding percentages can be a practical starting point while keeping in mind that this strategy will not always be reliable. Nevertheless, a thorough PK understanding can help minimize the incidence of antibiotic dosing variabilities and uncertain treatment responses [24]. In general, standard dosing regimens are acceptable for antibiotics like most beta-lactams that are hydrophilic with low protein binding. Variations in drug concentrations tend to arise, however, with medications that are either hydrophilic with higher protein binding or lipophilic with lower protein binding. In all cases, careful patient monitoring is key as these rules do not always apply, as evidenced by the current, ambiguous literature illustrating inconsistent drug sequestration in medications with a variety of PK characteristics. Table 3 summarizes therapy management recommendations based on a drug’s expected degree of drug sequestration. Given the nature of infections in critically ill patients, objective evidence for improvement may be difficult to interpret, but the signs and symptoms of infection (i.e., fever curve, white blood cell count, hemodynamic stability, culture results, etc.) can be used as part of the assessment of treatment efficacy. These should not be the only markers used, however. Therapeutic drug monitoring (TDM), when possible, represents the only true way of assessing the degree of drug sequestration and adequacy of serum concentrations. For antimicrobials with readily available monitoring, drug levels should be utilized consistently. For those agents where drug levels are not as readily available but still a possibility, TDM should still be considered in cases of severe infections or where there is a high concern for medication toxicity. In cases where TDM is non-standard or not easily obtained, efforts should be made to find alternative agents with less of a concern for drug sequestration, and, ideally, agents with TDM available. Managing antimicrobials in critically ill patients requiring ECMO support is difficult, but remembering general PK trends and their supposed influence on drug sequestration as well as utilizing all the available tools for monitoring can help maximize patient outcomes.

## Figures and Tables

**Table 1:** Expected Drug Sequestration Based on Medication PK Characteristics.

High Degree of Drug Sequestration		Low Suspicion for Drug Sequestration	
Lipophilic		Hydrophilic	
Highly protein bound		Low amount of protein binding	
Large molecular size		Smaller molecular size	
Ionized/charged molecules		Uncharged molecule	
**LogP and Protein Binding Characterization**			
Lipophilic LogP	≥1	High Protein Binding %	≥50%
Hydrophilic LogP	<1	Low Protein Binding %	<50%

Information adapted from: Ha et al. [2].

**Table 2:** Summary of Common ICU Antimicrobial Pharmacokinetic Characteristics.

	Lipophilic	Hydrophilic	Protein Binding
Ampicillin–sulbactam		+	30–40%
Cefazolin		+	85%
Ceftriaxone		+	95%
Metronidazole		+	20%
Piperacillin–tazobactam		+	30%
Cefepime		+	20%
Meropenem		+	2%
Ertapenem		+	95%
Imipenem–cilastatin		+	20–40%
Linezolid		+	30%
Vancomycin		+	50%
Daptomycin		+	90%
Micafungin		+	99%
Caspofungin		+	97%
Liposomal amphotericin B	+		90%
Amphotericin B deoxycholate		+	95%
Fluconazole		+	12%
Voriconazole	+		60%
Posaconazole	+		98%
Isavuconazole	+		99%

Information adapted from: Drugbank Online. https://go.drugbank.com/.

**Table 3:** Expected Degree of Drug Sequestration and Resulting Monitoring Recommendations.

	Low Protein Binding	High Protein Binding
LogP < 1	Low	Low
LogP ≥ 1	Low	High
**Monitoring Recommendations**		
Minimally Sequestered	Likely no dose adjustments necessary, utilize TDM when possible	
Highly Sequestered	Utilize TDM when possible (even with non-standard medications in situations with severe infections or high concern for medication toxicity) If TDM is non-standard or will be delayed, consider alternate antimicrobial agent with less concern for sequestration	

Information adapted from: Ha et al. [2].
